# Effect of TGF-β1 on eosinophils to induce cysteinyl leukotriene E_4_ production in aspirin-exacerbated respiratory disease

**DOI:** 10.1371/journal.pone.0256237

**Published:** 2021-08-26

**Authors:** Youngwoo Choi, Soyoon Sim, Dong-Hyun Lee, Hee-Ra Lee, Ga-Young Ban, Yoo Seob Shin, Yoon-Keun Kim, Hae-Sim Park

**Affiliations:** 1 Department of Allergy and Clinical Immunology, Ajou University School of Medicine, Suwon, Korea; 2 MD Healthcare Inc., Seoul, Korea; 3 Department of Pulmonology and Allergy, Hallym University Kangdong Sacred Heart Hospital, Seoul, Korea; Mohammed Bin Rashid University of Medicine and Health Sciences, UNITED ARAB EMIRATES

## Abstract

Cysteinyl leukotriene (cysLT) overproduction and eosinophil activation are hallmarks of aspirin-exacerbated respiratory disease (AERD). However, pathogenic mechanisms of AERD remain to be clarified. Here, we aimed to find the significance of transforming growth factor beta 1 (TGF-β1) in association with cysteinyl leukotriene E_4_ (LTE_4_) production, leading to eosinophil degranulation. To evaluate levels of serum TGF-β1, first cohort enrolled AERD (n = 336), ATA (n = 442) patients and healthy control subjects (HCs, n = 253). In addition, second cohort recruited AERD (n = 34) and ATA (n = 25) patients to investigate a relation between levels of serum TGF-β1 and urinary LTE_4_. The function of TGF-β1 in LTE_4_ production was further demonstrated by *ex vivo* (human peripheral eosinophils) or *in vivo* (BALB/c mice) experiment. As a result, the levels of serum TGF-β1 were significantly higher in AERD patients than in ATA patients or HCs (*P* = .001; respectively). Moreover, levels of serum TGF-β1 and urinary LTE_4_ had a positive correlation (*r* = 0.273, *P* = .037). In the presence of TGF-β1, leukotriene C_4_ synthase (LTC_4_S) expression was enhanced in peripheral eosinophils to produce LTE_4_, which sequentially induced eosinophil degranulation via the p38 pathway. When mice were treated with TGF-β1, significantly induced eosinophilia with increased LTE_4_ production in the lung tissues were noted. These findings suggest that higher levels of TGF-β1 in AERD patients may contribute to LTE_4_ production via enhancing LTC_4_S expression which induces eosinophil degranulation, accelerating airway inflammation.

## Introduction

Aspirin-exacerbated respiratory disease (AERD) typically presents moderate-to-severe phenotypes of asthma, chronic rhinosinusitis (CRS) and/or nasal polyps with persistent eosinophilia in the upper and lower airway mucosa. In addition, cysteinyl leukotriene (cysLT) overproduction is a hallmark of AERD, and the increased level of cysLTs derived from mast cells and eosinophils is a characteristic feature of AERD, in which leukotriene C_4_ synthase (LTC_4_S) is a key enzyme for converting arachidonic acid to cysLTs [1]. To date, type 1 cysteinyl leukotriene receptor (cysLT_1_R) antagonists and 5-lipoxygenase inhibitors have been used in the management of AERD [2]; however, there are unmet needs for its pathogenic mechanisms and therapeutic targets.

Eosinophilia in peripheral blood and upper/lower airway mucosa are commonly found in AERD patients [3]. Although both mast cells and eosinophils are critical for inducing airway inflammation in the pathogenesis of AERD, emerging evidence supports an important role of eosinophils in its pathogenesis [4,5]. AERD patients have shown that significantly elevated levels of eosinophil-derived granule proteins, such as eosinophil cationic protein (ECP) and eosinophil-derived neurotoxin (EDN), compared to aspirin-tolerant asthma (ATA) patients [6,7]. The direct effect of aspirin on eosinophil activation on releasing granule proteins has previously been demonstrated [8]. In addition, some studies have suggested that granule proteins play a crucial role in enhancing Th2 immune response among allergic diseases [9,10].

Transforming growth factor beta 1 (TGF-β1) has also been suggested to contribute to immune responses and structural changes in the lungs of asthmatic patients [11]. Moreover, this mediator is strongly expressed in nasal mucosa in response to inflammation, but not in normal nasal mucosa [12]. So far, the role of TGF-β1 in airway remodeling has mostly been highlighted; however, a few studies have shown that TGF-β1 down-regulates cyclooxygenase (COX)-2 in airway epithelial cells and then reduces prostaglandin E_2_ production [13]. Furthermore, enhanced LTC_4_S expression in fibroblasts and monocytes in the presence of TGF-β1 has been revealed [14,15], suggesting that TGF-β1 may contribute to cysLT production in AERD patients. Considering that persistent eosinophilic inflammation is a key feature of AERD and that studies evaluating the function of TGF-β1 in eosinophilic airway inflammation are still lacking, the present study focuses on the effect of TGF-β1 on eosinophil activation in AERD pathogenesis.

We hypothesized that TGF-β1 plays an important role in the severity of eosinophilic airway inflammation in AERD patients. This study compared the levels of serum TGF-β1 between AERD and ATA patients, investigated the association between TGF-β1 and LTE_4_ in the clinical cohort, and evaluated the effect of TGF-β1 on LTE_4_ production *ex vivo* or *in vivo*.

## Materials and methods

### Ethics

Two cohorts of adult asthmatic patients were assessed in this study approved by the Institutional Review Board of Ajou University Hospital (AJIRB-GEN-SMP-13-108; AJIRB-BMR-SUR-15-498). All patients provided written informed consent to participate in this study by signing the consent form.

### Patient cohorts, clinical parameters and serum cytokine levels

The clinical significance of TGF-β1 level in AERD was evaluated in which AERD (n = 336), ATA (n = 442) patients and healthy control subjects (HCs; n = 253) were recruited. Among AERD and ATA patients enrolled in the first cohort, we enrolled AERD (n = 34) and ATA (n = 25) patients who had wanted to participate voluntarily in the second cohort study to investigate the role of TGF-β1 in association with LTE_4_ metabolite levels/eosinophil activation markers. AERD were diagnosed according to clinical features previously described [16]. The diagnosis of AERD was based on a positive response to lysine-aspirin bronchoprovocation test (L-ASA BPT). The presence of CRS and nasal polyps was confirmed using paranasal sinus X-rays, CT scans and/or rhinoscopy as well as clinical symptoms. The degree of airway obstruction was evaluated using spirometry. The degree of airway hyperresponsiveness was examined by methacholine bronchial challenge test. Atopy status was defined as previously described [17]. The levels of serum IgE were quantified using UniCAP^®^ system (ThermoFisher Scientific, Waltham, MA, USA). The levels of serum TGF-β1 from every study subject were measured using ELISA (R&D systems, Minneapolis, MN, USA). Sputum collection and neutrophils/eosinophils counting were performed as previously described [18], which were always the same between the first and second cohorts. To determine TGF-β1-low/high groups, the cutoff value (48.1 ng/mL) was set at mean plus 2 standard deviations of the test values. In the second cohort, the urine and serum of each patient were simultaneously collected in the morning time during the enrollment period. The urinary LTE_4_ metabolite levels were measured using ultra-high-performance liquid chromatography system as previously described [19]. In addition, the levels of serum EDN were measured using the ELISA kit (SKIMS-BIO, Seoul, Korea).

### Stimulation of peripheral eosinophils from asthmatic patients

Peripheral eosinophils were isolated from asthmatic patients as previously described [20]. To stimulate eosinophils, the cells (1×10^6^) were seeded on a 24-well plate and maintained in RPMI-1640 medium (Sigma-Aldrich, St. Louis, MO, USA) supplemented with 2% fetal bovine serum (FBS; ThermoFisher Scientific). Then, the cells were treated with human recombinant 10 ng/mL IL-5 (Sigma-Aldrich) and 5 ng/mL TGF-β1 (R&D systems). To investigate the effect of cysLTs on eosinophil degranulation, the cells were treated with LTE_4_ (Cayman Chemical, Ann Arbor, MI, USA) for 4 hours in the presence of 10 ng/mL IL-5 (Sigma-Aldrich). The function of montelukast (Sigma-Aldrich; 0.1 and 1 μM) against LTE_4_ was also investigated. To confirm eosinophil degranulation, eosinophils were seeded on Poly L-lysine-coated slides (Polysciences, Warrington, PA, USA). Then the cells were incubated overnight with anti-eosinophil peroxidase antibody (Cell Signaling, Minneapolis, MN, USA), followed by Alexa fluor 488 donkey anti-rabbit (ThermoFisher Scientific) for 1 hour. 4’,6-diamidino-2-phenylindole (Sigma-Aldrich), and was observed using a Zeiss LSM710 confocal microscope (Carl Zeiss AG, Oberkochen, Germany).

### Interactions between eosinophils and airway epithelial cells

A549 cells (American Type Culture Collection, Manassas, VA, USA) were used to investigate the role of airway epithelial cells in eosinophilic airway inflammation. The cells (5×10^5^) were seeded on a 24-well plate in RPMI with 10% FBS. Then RPMI with 2% was used when A549 cells were treated with peripheral eosinophils (1×10^6^) from asthmatic patients for 24 hours. In addition, A549 cells were treated with EDN (Athens Research & Technology, Athens, GA, USA; 1, 10 and 100 ng/mL) for 24 hours to demonstrate the effect of granule proteins on airway epithelial cell stimulation. To collect supernatant, culture medium was centrifuged at 12,000 rpm for 20 min at 4°C.

### Polymerase chain reaction

Total RNA was isolated from human peripheral eosinophils using TRIzol^®^ (ThermoFisher Scientific), according to the manufacturer’s instructions. Then, 1 μg of total RNA was synthesized to the single-stranded cDNA using primers (LTC_4_S, Forward: 5’-AGGTGGGCTGGTTCCTATCTA-3’ and Reverse: 5′-CCCATGGCTATCCTACCATTT-3′; GAPDH, Forward: 5’-GCAAAGTCAAGGCTGAGAAC-3’ and Reverse: 5’-ATGGTGGTGAAGACGCCAGT-3’). The PCR products were separated by electrophoresis using a 1% ethidium bromide-stained agarose gel and visualized by ultraviolet transillumination.

### Western blot analysis

To separate proteins (total protein concentration of cell lysate; 50 μg), 10% sodium dodecyl sulphate–polyacrylamide gel electrophoresis was used. Then the gels were transferred to PVDF membrane (BIO-RAD, Hercules, CA, USA). The antibodies used were as follows: TGF-β1 receptor (TGFR1; Abcam, Cambridge, United Kingdom; 1:1,000; 45 kDa), TGF-β2 receptor (TGFR2; Abcam; 1:1,000; 75 kDa), LTC_4_S (Sigma-Aldrich; 1:500; 40 kDa), p38 (Cell Signaling Technology; 1:1000; 38 kDa), phospho-p38 (Cell Signaling Technology; 1: 500; 38 kDa), and actin (Santa Cruz, Dallas, TX, USA; 1:1,000; 42 kDa).

### *In vivo* mouse model

All experimental protocols were approved by the Institutional Animal Care and Use Committee of Ajou University (IACUC-2017-0067). Female 6-week-old BALB/c wild-type mice (Jackson Laboratory, Bar Harbor, ME, USA) were maintained under specific pathogen-free conditions. To demonstrate the effect of TGF-β1 on LTE_4_ production, mice (n = 6 mice per group) were intranasally injected with 0.1 μg of mouse recombinant TGF-β1 (R&D systems) for 5 days. Eosinophil numbers in bronchoalveolar lavage fluid (BALF) were determined by Diff-quick staining (Dade Behring, Dudingen, Switzerland). Moreover, LTE_4_ (MyBioSource, San Diego, CA, USA) and EDN (LifeSpan BioSciences, Seatle, WA, USA) in BALF were measured using ELISA kits.

### Statistical analysis

All statistical analyses were performed using IBM SPSS software, version 26.0 (IBM Corp., Armonk, NY, USA). *P* values < .05 was considered statistically significant. GraphPad Prism 8.0 software (GraphPad Inc., San Diego, CA, USA) was used to create graphs.

## Results

### Higher levels of serum TGF-β1 in AERD patients

Demographic data from the study subjects of the first cohort are described in Table 1. The presence of nasal polyps and decrease in FEV_1_ (%) after lysine-aspirin bronchoprovocation test were significantly higher in AERD patients than in ATA patients (*P* = .001 and *P* = .001, respectively). In addition, lower baseline FEV_1_ (%) and PC_20_ methacholine values were noted in the AERD patients compared to the ATA patients (*P* = .026 and *P* = .001, respectively), whereas total IgE, total eosinophil count and sputum eosinophil/neutrophils (%) were not significantly different between the 2 groups. However, the levels of serum TGF-β1 were significantly higher in the AERD patients than in the ATA patients (*P* = .001). When asthmatic patients were divided into the TGF-β1-low and -high subgroups (the cutoff value, 48.1 ng/mL), the TGF-β1-high subgroup showed lower baseline FEV_1_ (%) than the TGF-β1-low subgroup within the AERD group (*P* = .034), while no differences were found within the ATA group (Table 2). In this study, we enrolled the second cohort to verify the reproducibility of clinical data. As in the result of the first cohort, the levels of serum TGF-β1 were also significantly higher in the AERD group than in the ATA group (*P* = .026; Table 3). In addition, the levels of urinary LTE_4_ were significantly higher in the AERD group than those in the ATA group (*P* = .001; Table 3). These findings indicate that higher levels of TGF-β1 may have an important role in AERD pathogenesis.

**Table 1:** Demographic data from the study subjects enrolled in the first cohort of adult asthmatic patients.

Variables	AERD (n = 336)	ATA (n = 442)	HCs (n = 253)	*P* value		
				AERD vs.ATA	AERD vs. HCs	ATA vs. HCs
Age (y)	42.2 ± 13.9/336	44.7 ± 14.4/442	31.6 ± 10.6/253	.018	.001	.001
Female sex (%)	64.9/336	61.9/442	54.7/253	.385	.012	.065
Atopy (%)	52.1/330	48.8/412	28.0/200	.367	.001	.001
Nasal polyp (%)	42.1/271	18.2/148	NA	.001	NA	NA
Severe asthma (%)	22.5/329	16.8/440	NA	.048	NA	NA
Baseline FEV_1_ (%)	85.3 ± 20.2/316	88.8 ± 19.6/335	NA	.026	NA	NA
Fall of FEV_1_ (%)	16.1 ± 5.7/189	6.5 ± 3.8/185	NA	.001	NA	NA
PC_20_ (mg/mL)	3.2 ± 4.6/239	5.3 ± 5.7/246	NA	.001	NA	NA
Total IgE (kU/L)	358.4 ± 550.1/328	365.9 ± 590.0/410	74.0 ± 111.6/66	.857	.001	.001
TEC (/μL)	413.9 ± 412.2/310	384.0 ± 368.1/344	NA	.327	NA	NA
Sputum Eos (%)	23.9 ± 35.2/217	21.6 ± 32.5/216	NA	.470	NA	NA
Sputum Neu (%)	57.5 ± 34.6/166	59.6 ± 33.2/192	NA	.561	NA	NA
TGF-β1 (ng/mL)	33.1 ± 14.2/191	28.4 ± 15.7/304	22.5 ± 11.3/175	.001	.001	.021

*P* values were obtained by Pearson’s Chi-square test for categorical variables (sex, atopy, nasal polyp, severe asthma, baseline FEV_1_, Fall of FEV_1_, sputum Eos and Neu) and Student’s *t* test for continuous variables (age, PC_20_, total IgE, TEC, TGF-β1).

AERD, aspirin-exacerbated respiratory disease; ATA, aspirin-tolerant asthma; HCs, healthy control subjects; FEV_1_, forced expiratory volume in 1 s; PC_20_, the provocative concentration of methacholine required to cause a 20% fall in FEV_1_; Fall of FEV_1_; decrease in FEV_1_ after the inhalation of lysin aspirin; IgE, immunoglobulin E; TEC, total eosinophil count; Eos, eosinophils; Neu, neutrophils; NA, not available.

**Table 2:** Characteristics of asthmatic patients with high (≥48.1 ng/mL) and low TGF-β1 (<48.1 ng/mL) levels in the first cohort.

Variables	AERD		*P* value	ATA		*P* value
	High (n = 23)	Low (n = 168)		High (n = 27)	Low (n = 277)	
Age (y)	40.0 ± 13.8/23	43.9 ± 13.2/168	.192	41.5 ± 11.9/27	45.8 ± 14.7/277	.149
Female sex (%)	56.5/23	70.8/168	.164	59.3/27	60.3/277	.917
Atopy (%)	43.5/23	46.7/165	.774	54.2/24	50.0/256	.696
Nasal polyps (%)	57.1/21	47.5/118	.413	42.9/7	14.0/57	.056
Severe asthma (%)	30.4/23	29.3/167	.914	25.9/27	19.3/274	.414
Baseline FEV_1_ (%)	75.4 ± 19.2/21	85.5 ± 20.2/153	.034	82.5 ± 23.6/16	89.2 ± 20.0/207	.207
PC_20_ (mg/mL)	2.1 ± 2.7/16	3.5 ± 4.8/115	.278	7.2 ± 7.0/23	6.2 ± 6.2/255	.577
Total IgE (kU/L)	303.5 ± 285.5/23	363.4 ± 619.2/164	.649	553.8 ± 910.6/22	371.7 ± 599.0/255	.193
TEC (/μL)	352.3 ± 282.4/23	425.9 ± 410.2/150	.408	496.7 ± 370.4/13	368.1 ± 352.5/210	.204
Sputum Eos (%)	26.6 ± 39.5/19	21.1 ± 34.1/122	.520	24.5 ± 30.6/10	19.0 ± 32.1/141	.602
Sputum Neu (%)	58.0 ± 39.2/13	54.4 ± 34.2/95	.727	62.3 ± 29.8/9	62.7 ± 33.8/118	.973

*P* values were obtained by Pearson’s Chi-square test for categorical variables and Student’s *t* test for continuous variables.

**Table 3:** Demographic data of the study subjects enrolled in the second cohort.

Variables	AERD (n = 34)	ATA (n = 25)	*P* value
Age (y)	44.5 ± 10.3/34	49.2 ± 19.1/25	.266
Female sex (%)	70.6/34	76.0/25	.770
Atopy (%)	32.4/34	40.0/25	.544
Nasal polyp (%)	64.0/25	18.2/11	.014
Severe asthma (%)	52.9/34	32.0/25	.109
Baseline FEV_1_ (%)	86.6 ± 20.3/30	94.5 ± 15.3/15	.195
Fall of FEV_1_ (%)	17.8 ± 4.5/21	5.4 ± 2.1/9	.001
PC_20_ (mg/mL)	3.2 ± 4.3/25	4.8 ± 5.6/16	.308
Total IgE (kU/L)	232.7 ± 242.9/32	280.2 ± 312.8/20	.542
TEC (/μL)	493.3 ± 292.9/30	428.9 ± 280.5/16	.475
Sputum Eos (%)	30.8 ± 41.6/25	20.8 ± 32.3/15	.429
Sputum Neu (%)	36.6 ± 37.0/19	59.8 ± 34.0/14	.411
TGF-β1 (ng/mL)	36.9 ± 15.2/34	27.7 ± 15.3/25	.026
LTE_4_ (ng/mL creatinine)	0.4 ± 0.3/34	0.1 ± 0.2/25	.001

*P* values were obtained by Pearson’s Chi-square test for categorical variables and Student’s *t* test for continuous variables.

### Function of TGF-β1 in LTC_4_S expression and LTE_4_ production

**Fig 1:**
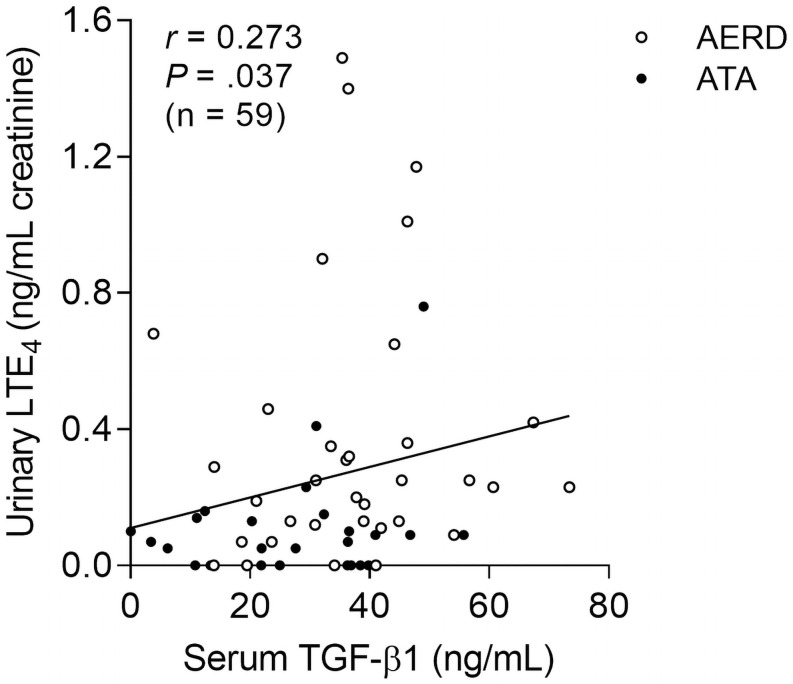
Association between levels of serum TGF-β1 and urinary LTE_4_ in the study subjects. Data are represented as Spearman correlation coefficient *r* (*P* value).

**Fig 2:**
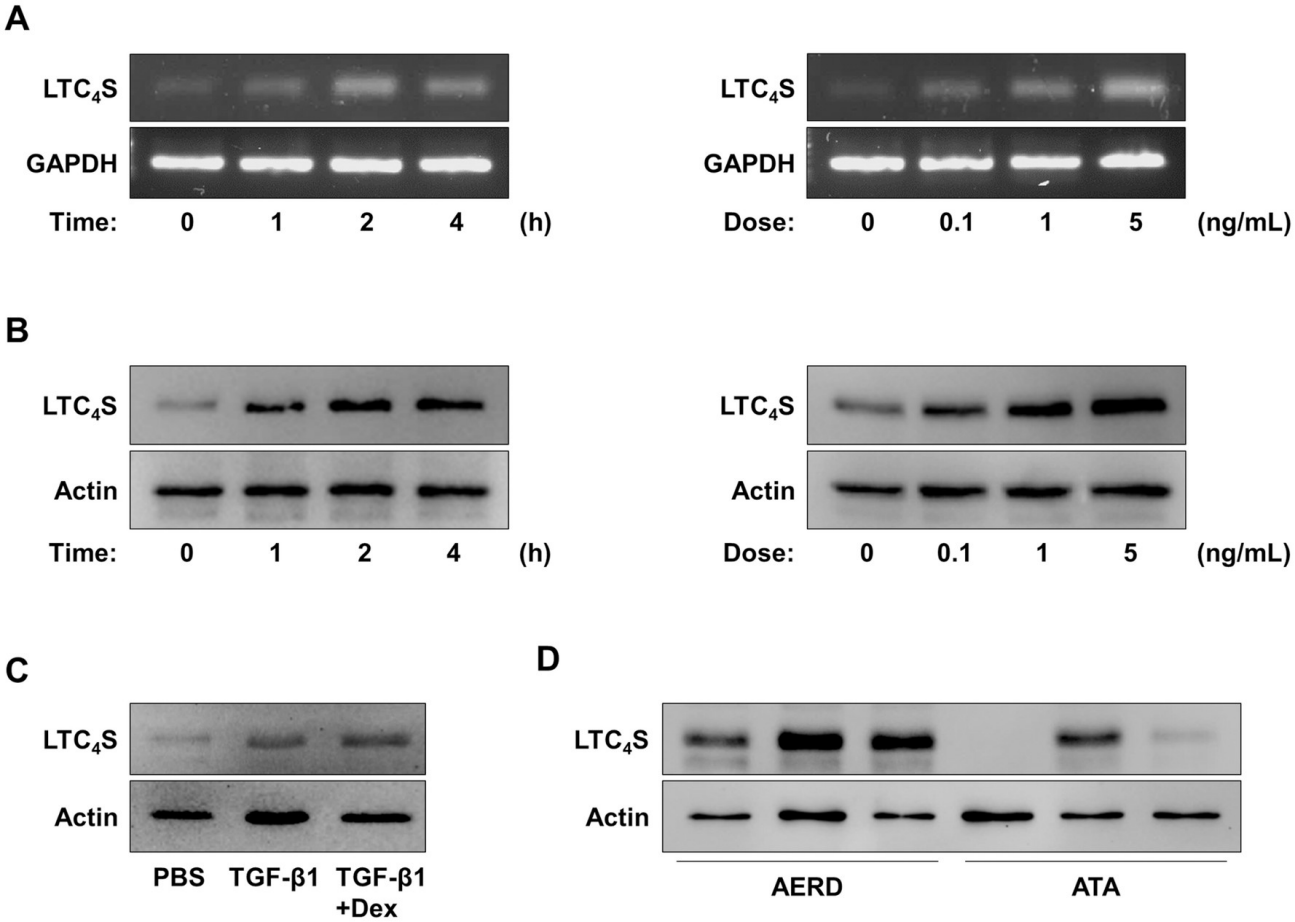
Effect of TGF-β1 on LTC_4_S expression in human peripheral eosinophils. Effect of TGF-β1 on **(A)** LTC_4_S expression and **(B)** LTC_4_S levels in peripheral eosinophils in a time- or dose-dependent manner (samples from 3 asthmatic patients were pooled). **(C)** Function of dexamethasone against TGF-β1 treatment (samples from 3 asthmatic patients were pooled). **(D)** Comparison of LTC_4_S levels between ARED and ATA patients (n = 3 asthmatic patients per group).

The levels of serum TGF-β1 and urinary LTE_4_ showed a significantly positive correlation (*r* = 0.273, *P* = .037; Fig 1). When peripheral eosinophils from asthmatic patients were treated with TGF-β1, expression of LTC_4_S in the cells was markedly upregulated (Fig 2A). In addition, levels of LTC_4_S in the cells were enhanced by TGF-β1 treatement (Fig 2B), but dexamethasone did not effectively reduce levels of LTC_4_S (Fig 2C). In the presence of TGF-β1, high levels of LTC_4_S with increased in peripheral eosinophils from the AERD patients was noted compared to those of the ATA patients (Fig 2D). Moreover, the TGF-β1 receptor (TGFR1) within eosinophils was highly expressed in peripheral eosinophils from the AERD patients than those from the ATA patients, while TGFR2 was not (S1 Fig). These results imply that eosinophils from AERD patients may be more sensitive to TGF-β1 in association with LTE_4_ production because of highly expressed TGFR1 on the surface of eosinophils.

### Induction of eosinophil degranulation by LTE_4_ treatment

**Fig 3:**
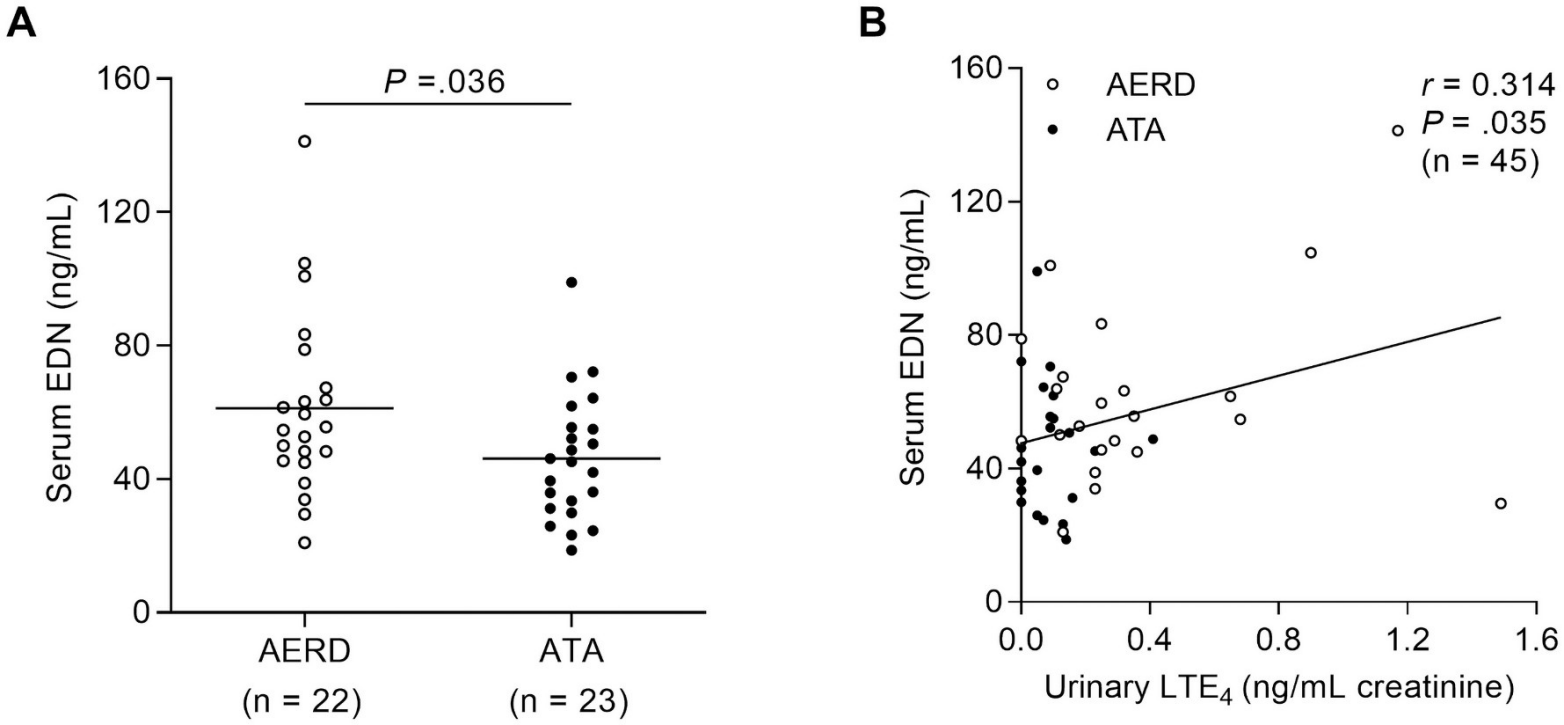
Relation between levels of urinary LTE_4_ and serum EDN. **(A)** Levels of serum EDN in the study subjects. Data are presented as mean. *P* values were obtained by Student’s *t* test. **(B)** A correlation between the levels of serum EDN and urinary LTE_4_. Data are represented as Spearman correlation coefficient *r* (*P* value).

**Fig 4:**
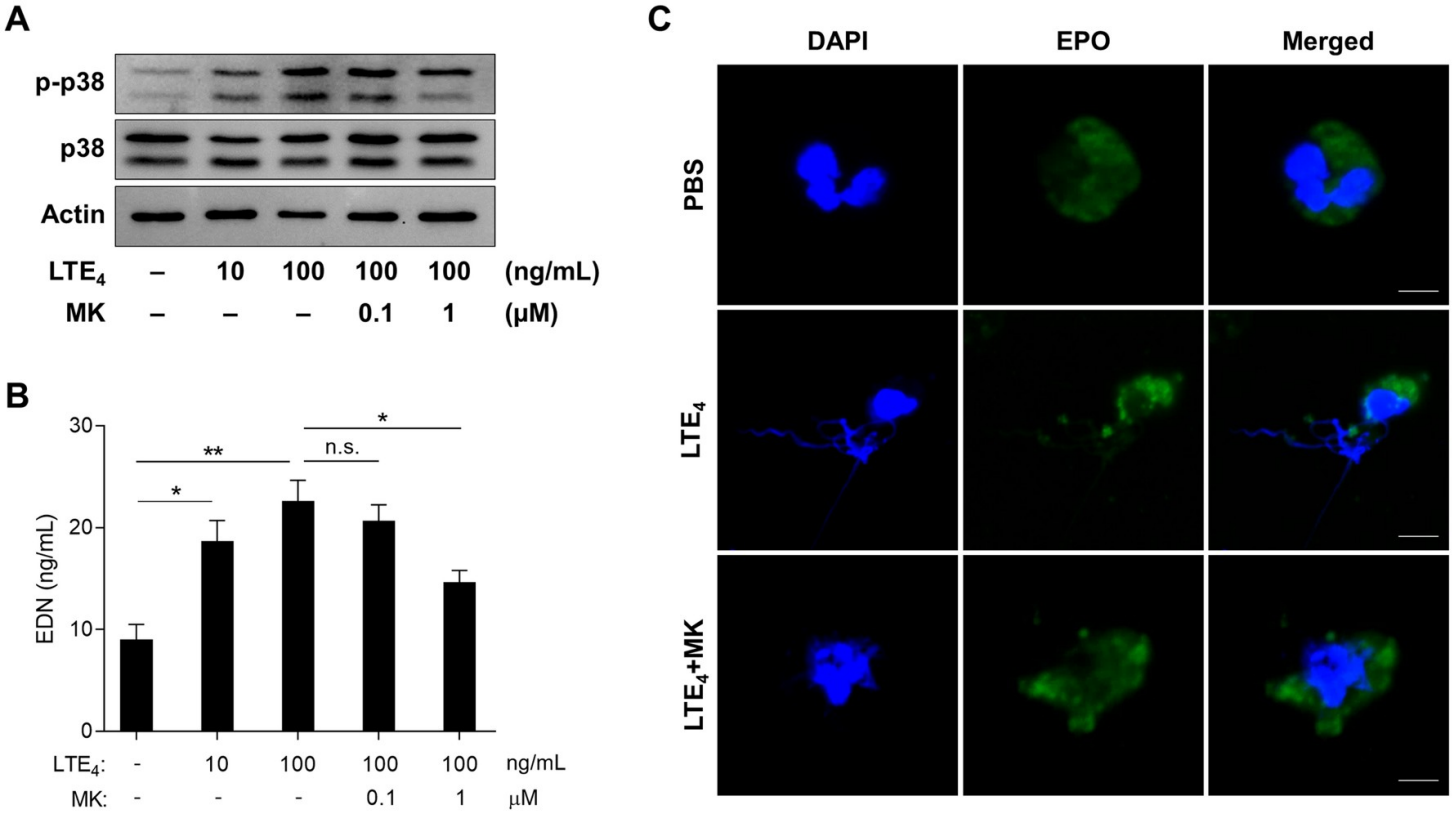
Function of LTE_4_ in eosinophil degranulation. **(A)** Phosphorylation of p38 in peripheral eosinophils (samples from 3 asthmatic patients were pooled). **(B)** Levels of EDN released from the cells. Data are presented as mean ± SD, n = 5. *P < .05 and **P < .01 were obtained by the Mann-Whitney test. n.s., not significant. **(C)** Images of eosinophils observed using confocal microscopy. Scale bar, 5 μm. DAPI, 4′,6-diamidino-2-phenylindole (blue); EPO, eosinophil peroxidase (green); MK, montelukast.

As TGF-β1 markedly enhanced LTE_4_ production by eosinophils, sequential effects of LTE_4_ on peripheral eosinophils were further investigated. In this study, we found significantly elevated levels of serum EDN in the AERD group compared to the ATA group (*P* = .036; Fig 3A). In addition, the levels of urinary LTE_4_ were positively correlated with serum EDN (*r* = 0.314, *P* = .035; Fig 3B). When peripheral eosinophils from asthmatic patients were treated with LTE_4_, phosphorylation of p38 was significantly elevated in the cells; however, montelukast (cysLT receptor 1 antagonist) could inhibit phosphorylation of signaling molecules (Fig 4A). In addition, LTE_4_ enhanced levels of EDN released from the eosinophils (Fig 4B). The eosinophils observed using confocal microscopy also showed granule proteins released by LTE_4_ stimulation (Fig 4C), indicating that LTE_4_ is important for inducing eosinophil degranulation through the p38 pathway.

### Effect of granule proteins in airway epithelial cells

When airway epithelial cells (A549 cells) were co-cultured with peripheral blood eosinophils with/without LTE_4_, significantly elevated levels of TGF-β1 in culture supernatant were noted; however, the effect of eosinophils in airway epithelial cells was partially attenuated by montelukast treatment (S2 Fig). In particular, EDN could enhance TGF-β1 production from airway epithelial cells. Although dexamethasone tends to inhibit the effect of granule proteins in airway epithelial cells, it could not fully attenuate TGF-β1 production from the cells (S3 Fig), suggesting limited action of corticosteroids against eosinophil granule proteins.

### Enhanced LTE_4_ and EDN production by TGF-β1 treatment *in vivo*

**Fig 5:**
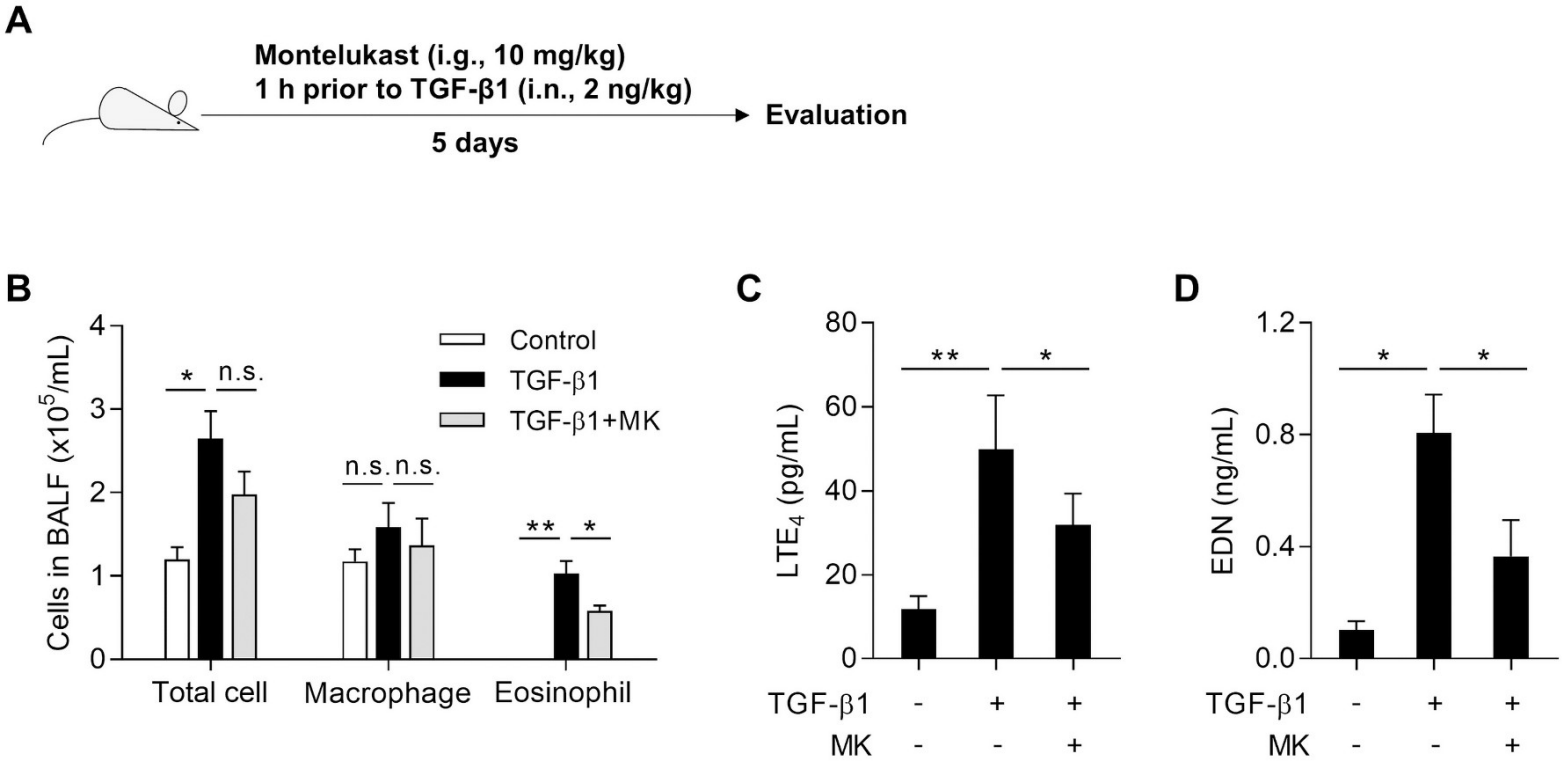
Roles of TGF-β1 in the lipoxygenase pathway to produce LTE_4_ *in vivo*. **(A)** Experimental schedule. **(B)** Differential cell count. Levels of **(C)** LTE_4_ and **(D)** EDN in bronchoalveolar lavage fluid. Data are presented as mean ± SD, n = 6. *P < .05 and **P < .01 were obtained by the Mann-Whitney test. n.s., not significant. i.n., intranasal injection; i.g., intragastric administration; MK, montelukast.

To demonstrate the effect of TGF-β1 on cysLT production, mice were intranasally injected with TGF-β1 with or without montelukast for every 5 days (Fig 5A). As a result, the total cell and eosinophil but not macrophage number in BALF was markedly elevated in mice treated with TGF-β1. Although montelukast did not fully reduce total cell count, the number of eosinophils was significantly decreased (Fig 5B). In addition, production of LTE_4_ and EDN was markedly elevated when mice were treated with TGF-β1, but montelukast could attenuated these mediators (Fig 5C and 5D). These findings show that TGF-β1 may contribute to eosinophilic airway inflammation through induction of LTE_4_ production.

## Discussion

This is the first study to demonstrate the pathophysiological function of TGF-β1 in AERD in the 2 clinical cohorts of adult asthmatic patients. It was found that the levels of serum TGF-β1 were higher in the AERD patients than in the ATA patients. AERD patients with higher TGF-β1 levels had lower FEV_1_ (%) and PC_20_ methacholine values, suggesting that TGF-β1 may be involved in the lung. In addition, *ex vivo* and *in vivo* studies confirmed an association between TGF-β1 and cysLT overproduction in AERD pathogenesis. Furthermore, increased LTE_4_ could induce eosinophil degranulation, which further stimulates airway epithelial cells to produce TGF-β1, resulting in the formation of the vicious circle. These provide a new insight into AERD pathogenesis via the TGF-β1-LTE_4_-eosinophil axis.

In the present study, significantly elevated levels of serum TGF-β1 were noted in the AERD patients compared to the ATA patients having a positive correlation with the levels of urinary LTE_4_. Previously, TGF-β1 polymorphisms have been suggested as a risk factor for AERD development, and TGF-β1 was associated with the prevalence of CRS in AERD patients, but not in ATA patients [21]. Nevertheless, the function of TGF-β1 in the pathogenesis of AERD has not been fully understood. Therefore, we aimed to find the functional effect of TGF-β1 in eosinophils, especially LTE_4_ production and eosinophil activation. Although the role of TGF-β1 in the arachidonic acid pathway was not well studied, a previous study revealed that TGF-β1 contributed to changes in LTC_4_S expression and cysLT_1_R in astrocytes [22]. Our *ex vivo* study demonstrated significantly enhanced LTC_4_S expression and LTE_4_ production in response to TGF-β1 in peripheral eosinophils from asthmatic patients. These suggest that TGF-β1 may be an essential factor for LTE_4_ production.

Here, we found highly expressed TGFR1, but not TGFR2 in peripheral eosinophils from AERD patients than from ATA patients. Previously, TGF-β1 has been shown to enhance the expression of TGFR1, and activation of Smad and MAPK/ERK in fibroblasts [23]. These findings are one of the plausible mechanisms explaining how eosinophils could produce more LTE_4_ in association with an increased level of TGF-β1 in AERD patients. In addition, previous studies have shown that AERD patients present moderate to severe phenotypes with lower levels of FEV_1_ (%) and PC_20_ methacholine compared to ATA patients [24,25]. Here, we also showed that AERD patients with higher levels of serum TGF-β1 with lower levels of FEV_1_ (%), suggesting that TGF-β1 may contribute to presenting more severe phenotypes with lung dysfunction. As conventional anti-inflammatory medications have limited effects in the TGF-β1-mediated inflammatory pathway [2], a new therapeutic strategy to suppress the pathway noted in the present study is required in long-term management of AERD.

Overproduction of cysLTs is the key finding in the pathogenesis and progression of AERD pathogenesis. In particular, LTE_4_ is involved in persistent eosinophilia via enhancement of eosinophil recruitment to the airway mucosa and bronchoconstriction [26]. In this study, LTE_4_ could stimulate eosinophils to secrete EDN through p38 phosphorylation, similar to eotaxin (a potent stimulator of eosinophil chemotaxis) which binds to a CC chemokine receptor and induces eosinophil degranulation through activation of the ERK–p38 pathway [27]. However, LTE_4_ certainly activates eosinophils via cysLT_1_R rather than other receptors as montelukast reduced levels of granule proteins released from eosinophils. A previous paper has also been shown that LTE_4_ is able to release granule proteins through binding to cysLT_1_R (major) and other receptors (minor) [28]. These implicate that increased levels of LTE_4_ may be responsible for enhancing eosinophil degranulation as well as eosinophil activation/recruitment, exacerbating type 2 airway inflammation in AERD patients where leukotriene receptor antagonists have partially suppressive effects.

Increased eosinophil number and activation markers in blood, sputum and tissues are common characteristics of bronchial asthma. The eosinophils communicate with several cell types involved in the pathogenesis of asthma; however, eosinophil-epithelial cell interactions have been extensively highlighted to play an important role in the processes of chronic airway inflammation as airway epithelial cells are a regulator of both innate and adaptive immune responses to host defence [29,30]. Following stimulation by multiple factors, airway epithelial cells produce large quantities of cytokines, chemokines and growth factors, such as TGF-β1, enhancing type 2 immune response [31,32]. Although the mechanism of TGF-β1 production from airway epithelial cells has not been fully elucidated, eosinophil granule proteins, such as ECP and EDN, are a possible factor contributing to the stimulation of the airway [33–35]. The function of EDN in enhancing airway remodeling in patients with eosinophilic CRS has been shown [36]. Furthermore, a recent study in adult asthmatic cohorts demonstrated that higher serum EDN was associated with severe asthma with asthma exacerbation [37]. In addition, a novel effect of EDN in airway epithelial cells on releasing TGF-β1 was noted in our *in vitro* study, where steroid may have a limited action. Taken together, these findings provide a possible mechanism of how activated eosinophils to induce TGF-β1 production contributing to chronic progressive type 2 airway inflammation in AERD pathogenesis.

This study has some limitations. First, the effect of TGF-β1 in multiple cells, such as mast cells, neutrophils or platelets, has not been determined. Secondly, further clinical trials in AERD patients according to the results of serum TGF-β1 levels and eosinophil activation status are needed to validate our findings.

In conclusion, TGF-β1 has a novel function contributing to cysLT overproduction through induction of LTC_4_S expression in eosinophils of AERD patients. Moreover, increased LTE_4_ induces eosinophil degranulation via the p38 pathway which further stimulates airway epithelial cells, suggesting that TGF-β1 plays a key role in enhancing eosinophilic airway inflammation, leading to poor clinical outcomes of AERD patients.

## Supporting information

S1 Fig Expression of TGF-β1 and TGF-β2 receptors in human peripheral eosinophils. (n = 3 asthmatic patients per group) TGFR1, TGF-β1 receptor; TGFR2, TGF-β2 receptor.
(PDF)

S2 Fig Effect of eosinophils on airway epithelial cells by secreting granule proteins. Levels of TGF-β1 released from A549 cells when co-cultured with peripheral eosinophils with/without LTE4 or montelukast (MK). The data are presented as means ± SD, n = 5. *P < .05 and **P < .01 were obtained by the Mann-Whitney test. n.s., not significant.
(PDF)

S3 Fig Function of dexamethasone (Dex) against eosinophil granule proteins to suppress TGF-β1 production from airway epithelial cells. The data are presented as means ± SD, n = 5. *P < .05 was obtained by the Mann-Whitney test. n.s., not significant. EDN, eosinophil-derived neurotoxin.
(PDF)
