# Supplementary material for: Effect of TGF-β1 on eosinophils to induce cysteinyl leukotriene E4 production in aspirin-exacerbated respiratory disease
Source: PLoS One. 2021 Aug 26;16(8):e0256237. doi: 10.1371/journal.pone.0256237 (PMC8389430; doi:10.1371/journal.pone.0256237)
Supplement: S1 Fig — (n = 3 asthmatic patients per group) TGFR1, TGF-β1 receptor; TGFR2, TGF-β2 receptor. (PDF) [file pone.0256237.s001.pdf]

Supplementary Figures

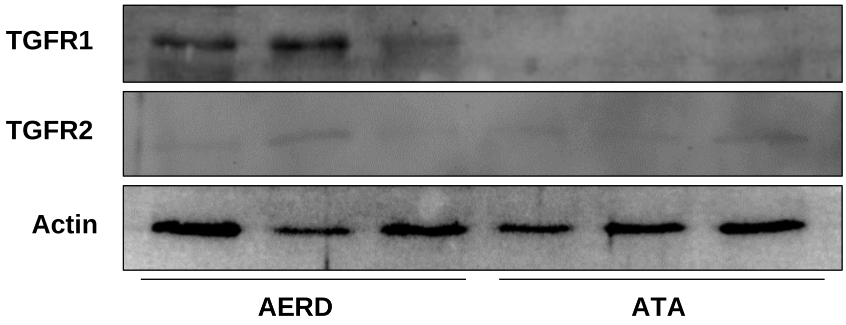

S1 Fig. Expression of TGF-β1 and TGF-β2 receptors in human peripheral eosinophils. (n = 3 asthmatic patients per group) TGFR1, TGF-β1 receptor; TGFR2, TGF-β2 receptor.
