# Supplementary material for: Effect of TGF-β1 on eosinophils to induce cysteinyl leukotriene E4 production in aspirin-exacerbated respiratory disease
Source: PLoS One. 2021 Aug 26;16(8):e0256237. doi: 10.1371/journal.pone.0256237 (PMC8389430; doi:10.1371/journal.pone.0256237)
Supplement: S2 Fig — Levels of TGF-β1 released from A549 cells when co-cultured with peripheral eosinophils with/without LTE4 or montelukast (MK). The data are presented as means ± SD, n = 5. *P < .05 and **P < .01 were obtained by the Mann-Whitney test. n.s., not significant. (PDF) [file pone.0256237.s002.pdf]

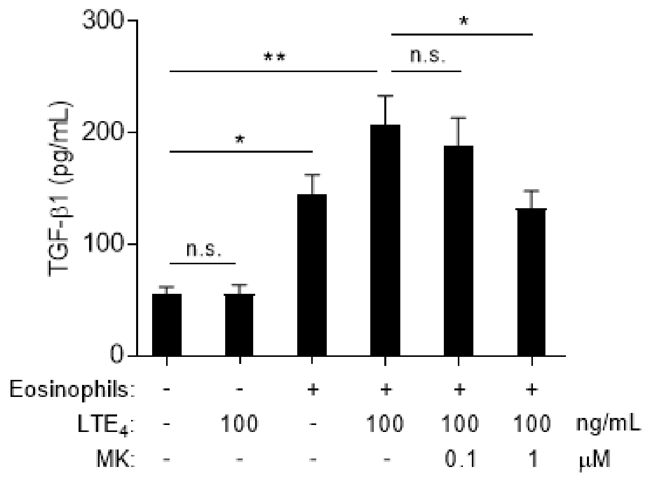

S2 Fig. Effect of eosinophils on airway epithelial cells by secreting granule proteins. Levels of TGF-β1 released from A549 cells when co-cultured with peripheral eosinophils with/without LTE_4 or montelukast (MK). The data are presented as means ± SD, n = 5. **P* < .05 and ***P* < .01 were obtained by the Mann-Whitney test. n.s., not significant.
