# Supplementary material for: Effect of TGF-β1 on eosinophils to induce cysteinyl leukotriene E4 production in aspirin-exacerbated respiratory disease
Source: PLoS One. 2021 Aug 26;16(8):e0256237. doi: 10.1371/journal.pone.0256237 (PMC8389430; doi:10.1371/journal.pone.0256237)
Supplement: S3 Fig — The data are presented as means ± SD, n = 5. *P < .05 was obtained by the Mann-Whitney test. n.s., not significant. EDN, eosinophil-derived neurotoxin. (PDF) [file pone.0256237.s003.pdf]

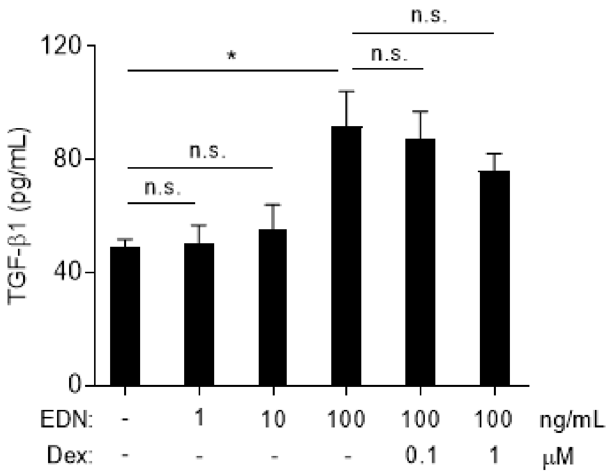

S3 Fig. Function of dexamethasone (Dex) against eosinophil granule proteins to suppress TGF-β1 production from airway epithelial cells. The data are presented as means ± SD, n = 5. **P* < .05 was obtained by the Mann-Whitney test. n.s., not significant. EDN, eosinophil-derived neurotoxin.
